# Supplementary material for: Megakaryocyte- and erythroblast-specific cell-free DNA patterns in plasma and platelets reflect thrombopoiesis and erythropoiesis levels
Source: Nat Commun. 2023 Nov 20;14:7542. doi: 10.1038/s41467-023-43310-2 (PMC10662131; doi:10.1038/s41467-023-43310-2)
Supplement: Supplementary file 1 — Supplementary Information [file 41467_2023_43310_MOESM1_ESM.pdf]

Supplementary Tables

Tissue	Marker	Genome location (hg19)	Primer 1	Primer 2
Leukocyte	PTPRCAP	chr11:67205010	GTTTTATAGGTTTAGATATTTTAGTTT	CAACCAAAACTAAAAATAAATACC
Leukocyte	SNX1	chr17:46190650	TTTTATGTATAGATTAATAGTAAAGTTTT	AAACCAACATTTCTCTATAACTACT
Liver	IGF2R	chr6:160500566	TGGGTGTTGTTATTTTGTTGA	CTACAAAAATACACACCCCAA
Liver	ITIH4	chr3:52864973	ATAGTGAAGATGTTAGTTTGTTTTT	AACACACTTACCTAATAACCAAAC
Liver	VTN2	chr17:26696304	GGTATTTTGAAGAGGTAGGTTT	ACCTAAATACCCCAAACTCAT
Liver	GPAM	chr10:113943113	TTTTTTATTGTTTTAATGTTTTTTAG	TAAACTCAATCCCCTAAATATCTAC
Liver	cg17952661	chr1:145395716	AGTTTTTTTATAATAGTTTTTTGTTAT	ACACTAAAATTTCAAACAAAACTC
MK	MK1	chr21:47808908	GTTTTGGTTTTTTGTTAGTTTT	AAAAAACCACAAAACTCAAAA
MK	MK2	chr21:46283807	AGAAGAGTTTTTAAGAATATGGTTT	ATATTCTTAAAAACTCTTCTTCCTC
MK	MK3	chr7:101560256	ATATTTGTTAGTTTGGTTTTTATGG	AAAAAATATAAAAAAAACTAAAATCC
Erythro.	eryth2	chr1:1197563	TCTTCAGATCAGCTGAAGGCACACACA	GATGATGGAGGGCCCACCCTA
Erythro.	eryth3	chr5:140058542	AGCCCCAAGTAACTCATCCTGCCCCA	GTTTAATGTTGGTCAGTTTATTCAT
Erythro.	RITA1	chr12:113629243	ACCACCTACCCCCAGGGGTAGCCACT	GGCAGACTCCCAGAAGTTATCTATGG
Erythro.	SPIDR	chr8:48469824	GGTGACCTTTGGGTAGTCAATG	GCCTGTCCAGGCCAGTTGCTGCACC
Erythro.	OSBP2	chr22:31094074	GACTGAGCATCCTCCAGGTCTGTCA	GTGCATGTGTGTTATCTCCTCCAGCC

Table S1. Primer sequences for amplification of cell-type specific markers for targeted bisufite-sequencing analysis.

	% Megakaryocyte DNA	DNA concentration in platelet+plasma (ng/ml)
SDP 001	85.69	44.55
SDP 588	89.96	6.96
SDP 593	72.87	11.61
SDP 758	80.88	4.18
SDP 758 DNase	90.84	13.00
SDP 1024	82.88	3.94
SDP 1036	69.52	11.28
SDP 1037	91.55	14.44
SDP 1257	66.24	0.73
SDP 1257 DNase	67.97	2.45
SDP 1263	79.31	0.64
SDP 1263 DNase	86.72	1.43
SDP 1301	83.25	1.12
SDP 1301 DNase	92.23	1.97
SDP 1302	74.41	2.59
SDP 1302 Dnase	88.59	3.42
Average ng/ml	7.77	
Average genomes/ml	2353.74	
Average genomes/200 ml platelet bag	470748.38	
Average genomes per platelet (assuming 2.5x10^11 platelets per bag)	1.883E-06	
Expected genomes per platelet (assuming 4000 platelets per MK, and n=12 MK ploidy)	0.003	
% of expected (% of MK DNA in platelets)	0.06277	

Table S2. Concentration of DNA and DNA methlyation analysis of SDP platelet preps.

Group	n	Age (avg.)	Age (SD)	Female (n)	Platelet count (/ul, avg.)	Platelet count (/ul, min)	Platelet count (/ul, max)	MK cfDNA (GE/ml)	Erythro. cfDNA (GE/ml)
ITP	5	42.0	24.5	3	14400	2000	33000	1608.2	20.8
ET	5	67.4	15.4	3	1064600	736000	1508000	4242.4	112.0
Hypoplastic BM	5	60.3	21.1	2	39600	8000	72000	249.5	2125.7
Healthy	77	36.5	14.6	40	302545	157000	494000	510.2	6.6
Leukocytes	41	33.0	14.6	13	NA	NA	NA	NA	NA
Thalassemia	3	47.7	7.1	2	619667	519000	749000	690.1	1657.9
Platelet Transfusion	11	64.7	11.0	11	19000	3000	47000	4005.1	1921.9

Table S3. Summary statistics of samples in study.

Supplementary Figures

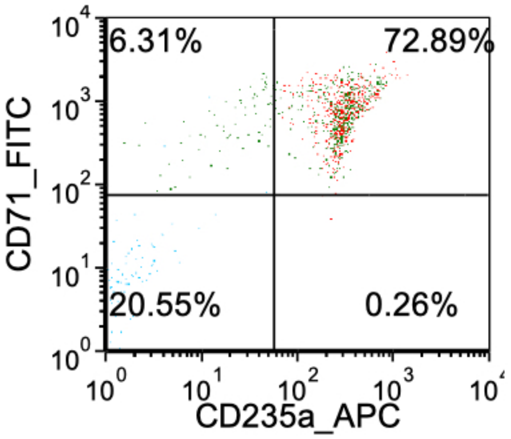

Figure S1. FACS plot of erythroid cells sorted from bone marrow. CD45-negative, CD235a and CD71-positive erythrocyte precursors, derived from bone marrow were FACS-sorted on a BD FACSAriaTM III flow cytometer.

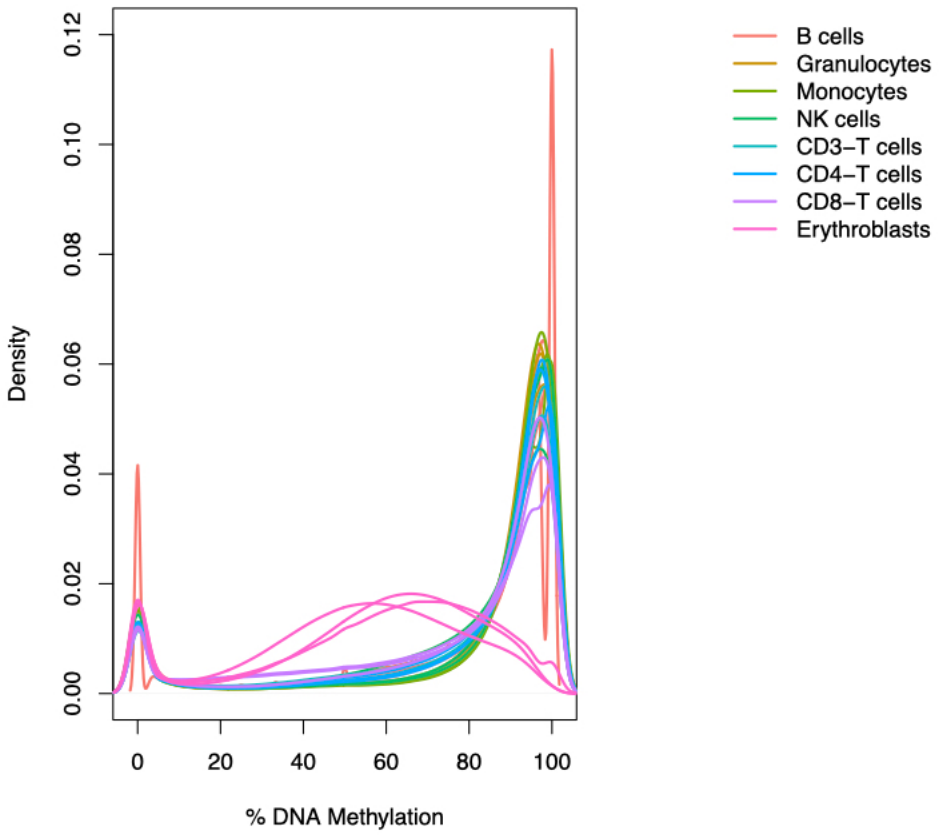

Figure S2. Density plot of methylomes of hematopoietic lineage demonstrating global demethylation in erythroblasts as compared to other cell types. Using whole-genome bisulfite sequencing (WGBS) data from Loyfer et al.[1], density plots of percent DNA methylation of CpGs across the entire genome were produced for replicates of cell types of hematopoietic origin.

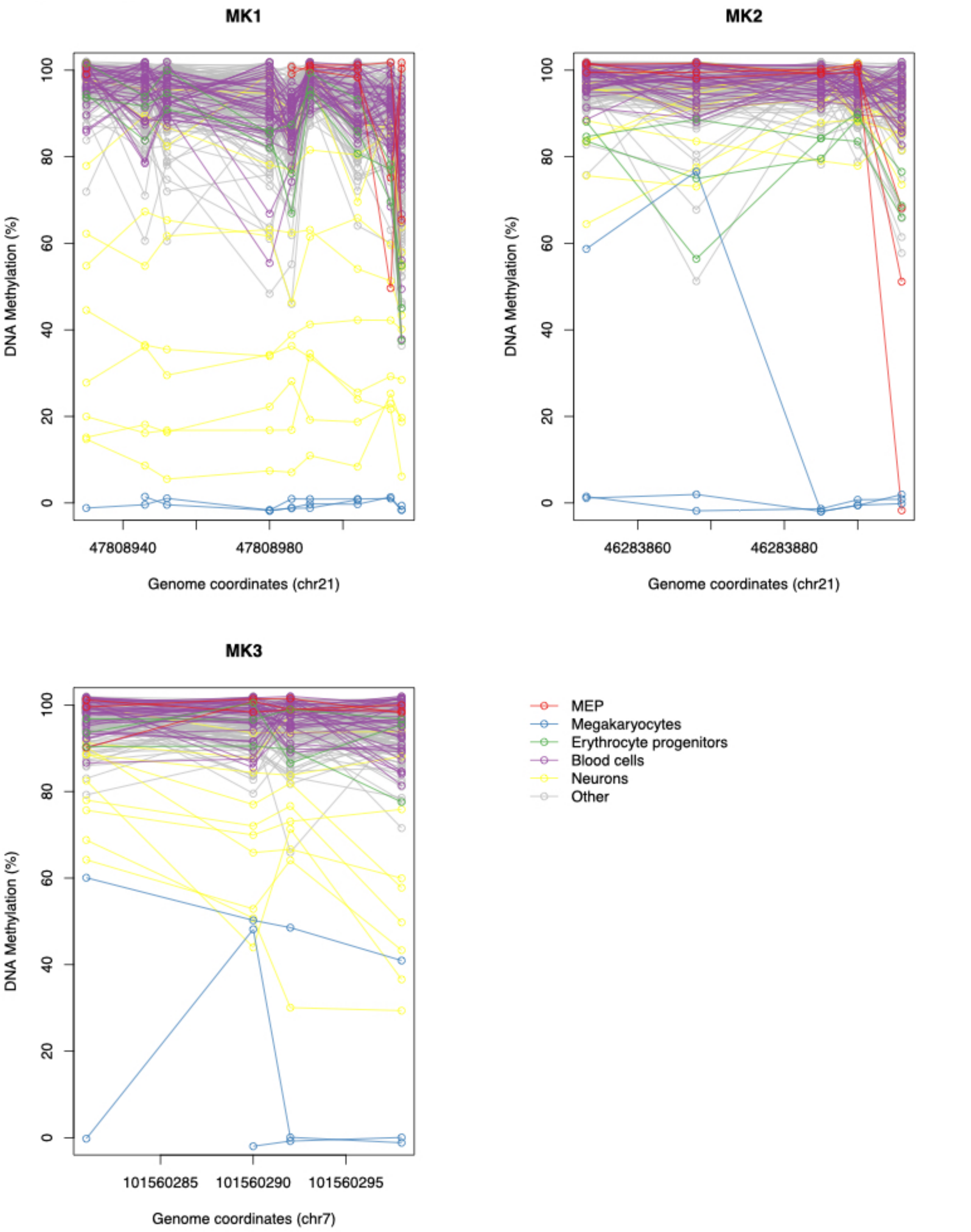

Figure S3. Megakaryocyte DNA methylation markers in megakaryocytes and other cell types. Percent DNA methylation at megakaryocyte-specific marker regions used for targeted bisulfite sequencing are analyzed using WGBS data at these regions in multiple cell types. Details of cell types analyzed are available in source data file.

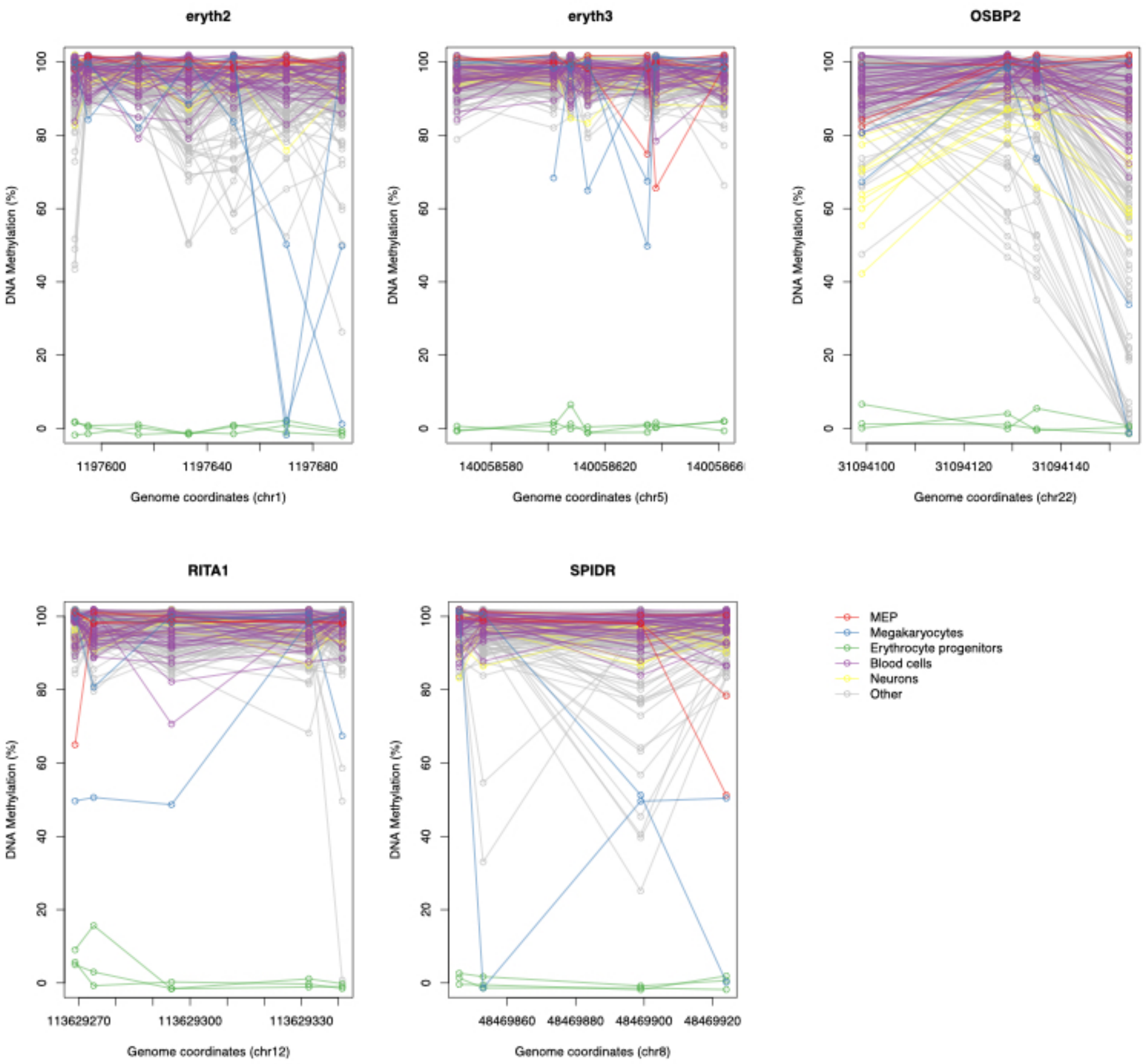

Figure S4. Erythroblast DNA methylation markers in megakaryocytes and other cell types. Percent DNA methylation at erythroblast-specific marker regions used for targeted bisulfite sequencing are analyzed using WGBS data at these regions in multiple cell types. Details of cell types analyzed are available in source data file.

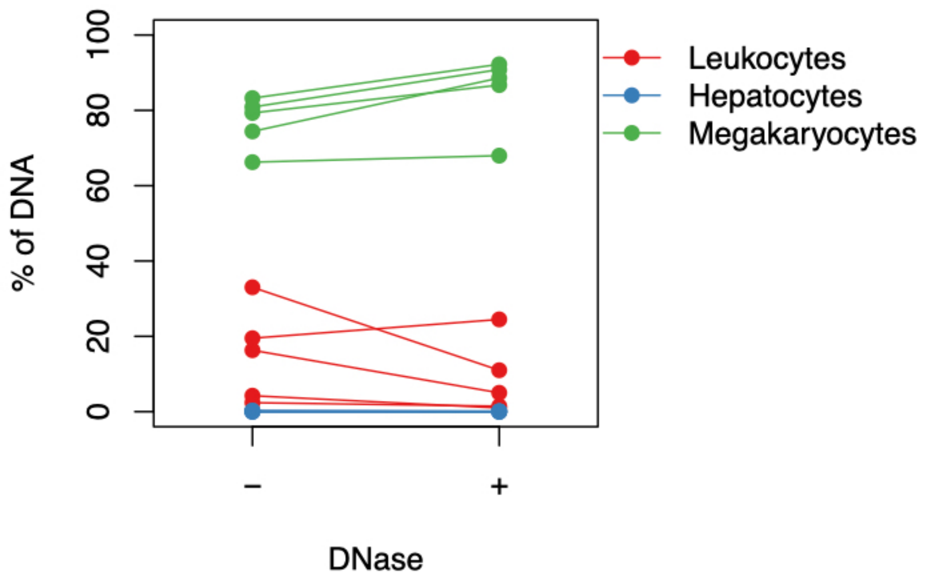

Figure S5. MK DNA in centrifuged platelets treated with DNase.
The percentage of MK DNA isolated from centrifuged platelets is significantly increased when treating with DNase, supporting the presence of MK DNA within platelets as opposed to leukocyte and liver DNA (n=5, $p<0.05$, paired two-tailed Mann-Whitney test).

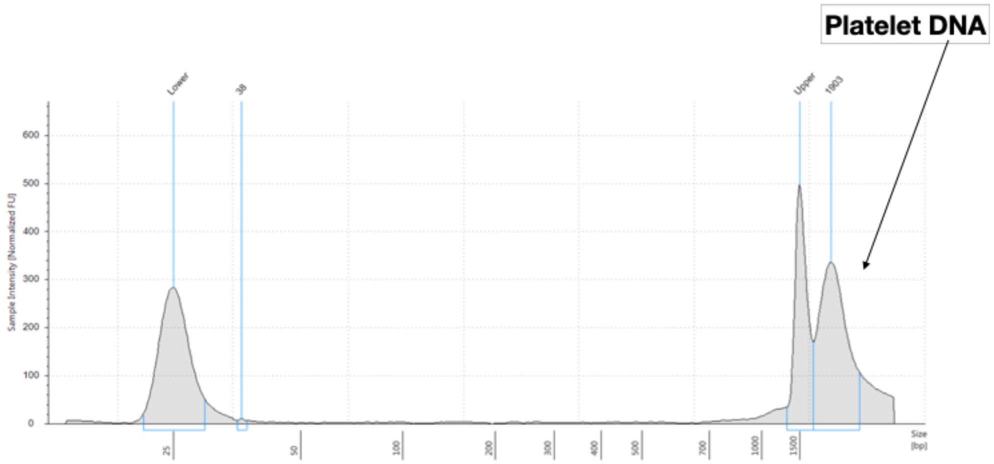

Figure S6. Platelet DNA is composed of high molecular weight DNA, as measured by Agilent Tapestation High Sensitivity D1000 system. DNA from one platelet sample was extracted and size was analyzed using the Tapestation system, demonstrating a significant peak of DNA larger than range for this system (0-1000 bp), indicative of high molecular weight DNA.

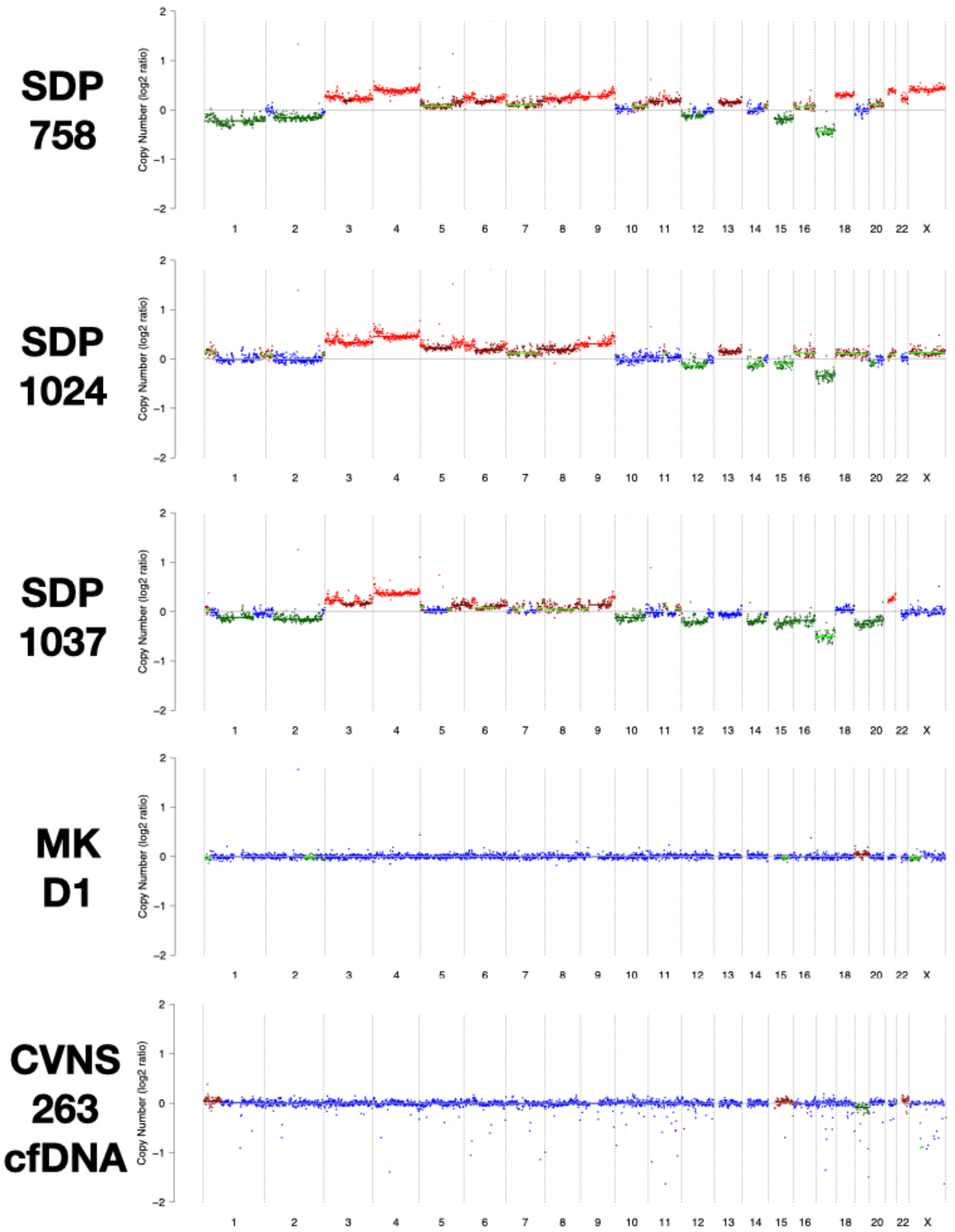

Figure S7. Copy number aberrations, calculated by ichorCNA for 3 individual platelet samples, megakaryocytes, and cfDNA, demonstrating marked copy number changes in platelet DNA. WGBS reads for platelet, megakaryocyte and cfDNA samples were binned into 1 MB size regions and normalized copy number change was calculated, demonstrating copy number changes in all 3 platelet samples analyzed.

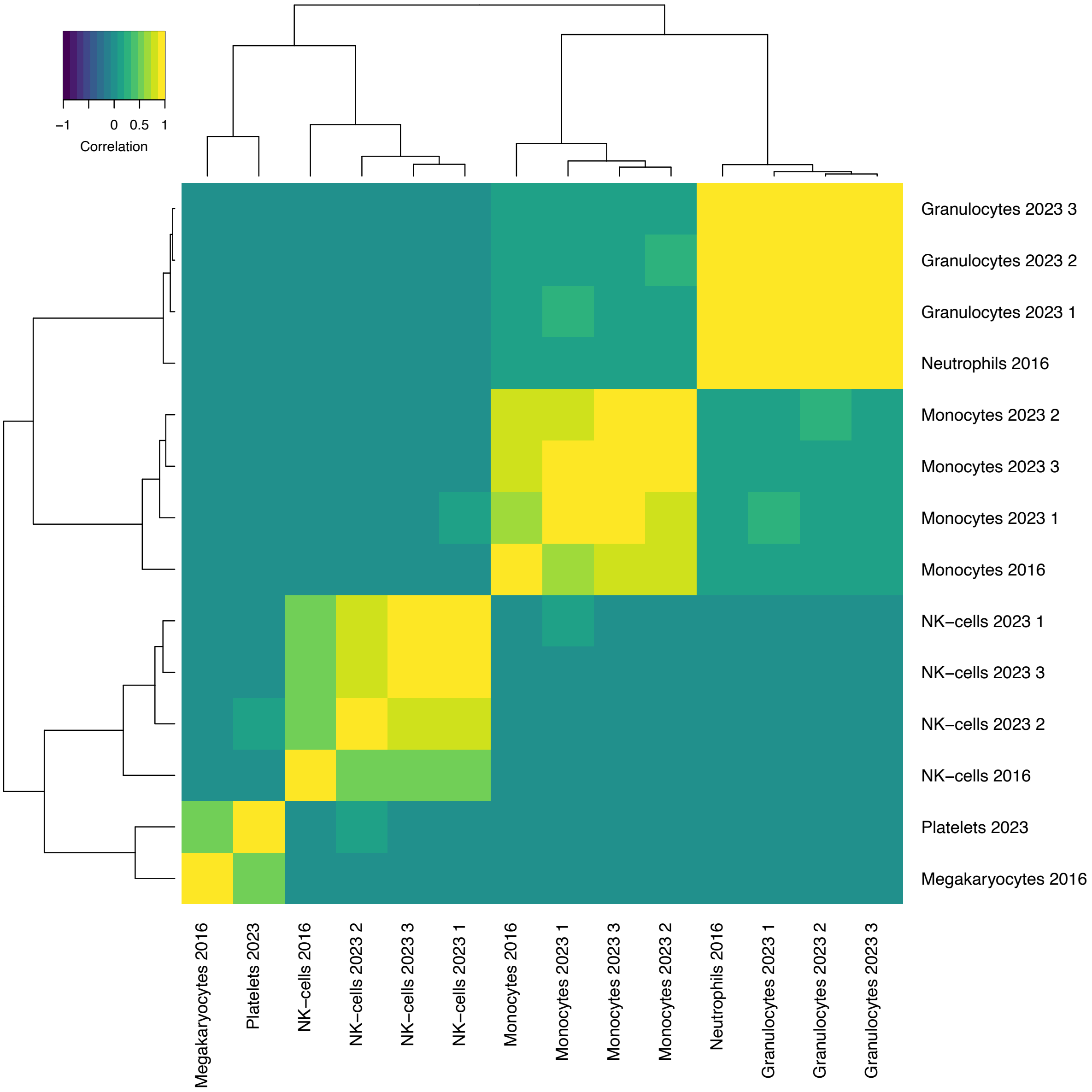

Figure S8. Comparability of datasets. Across cell type specific marker regions, platelet DNA is highly correlated to MK DNA and samples of different cell types for which markers were identified using data published in 2023 (Loyfer et al.[1]) correlate with samples published in 2016, supporting comparability of datasets produced in different studies.

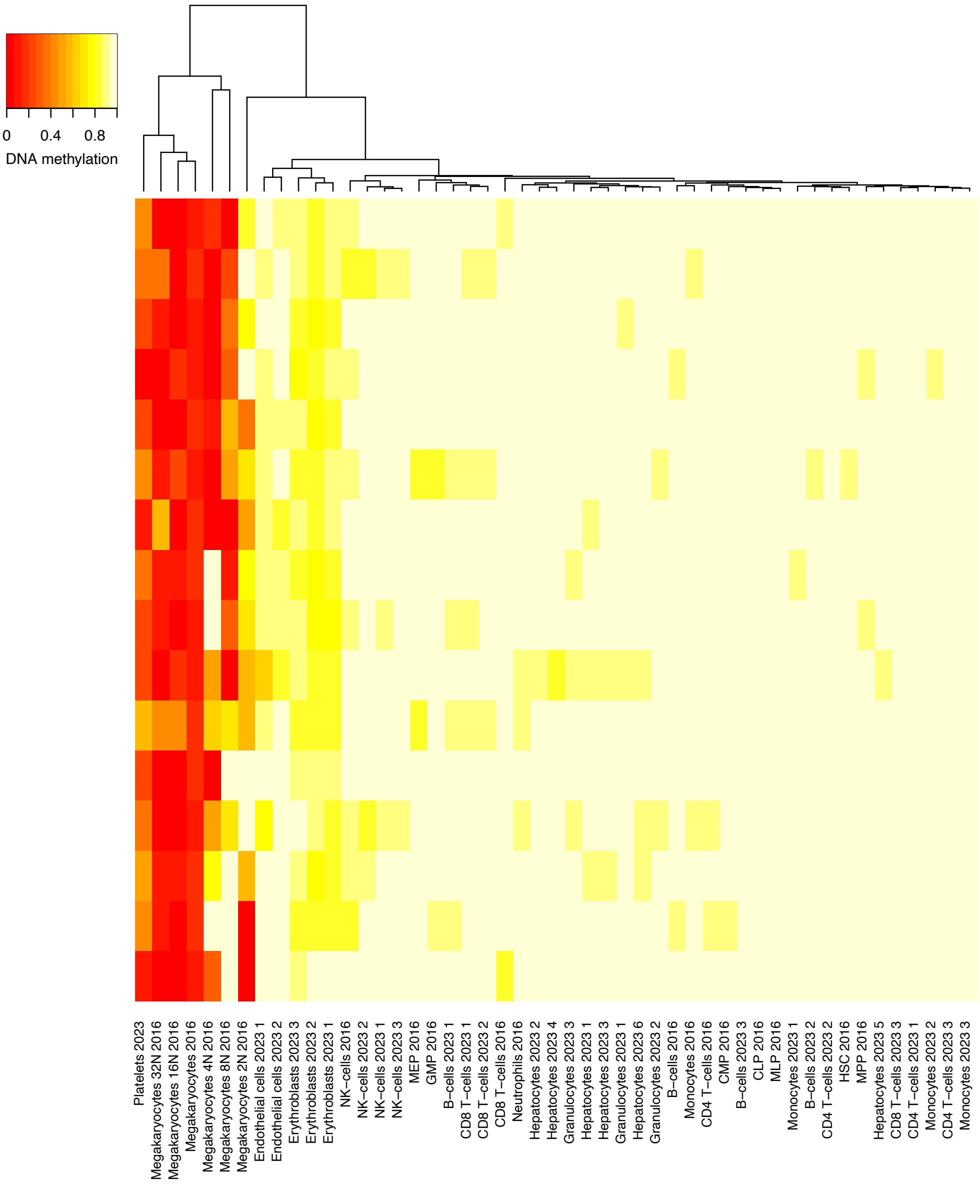

Figure S9. Effect of ploidy on methylation markers. Megakaryocyte (MK) DNA methylation data was separated by ploidy, and regions uniquely unmethylated in MK were analyzed in MK samples and samples of other cell types. Some of the regions appear to be more methylated in cells of lesser ploidy. Yet, methylation of these regions clearly distinguishes all MK (and platelet) samples from other samples.

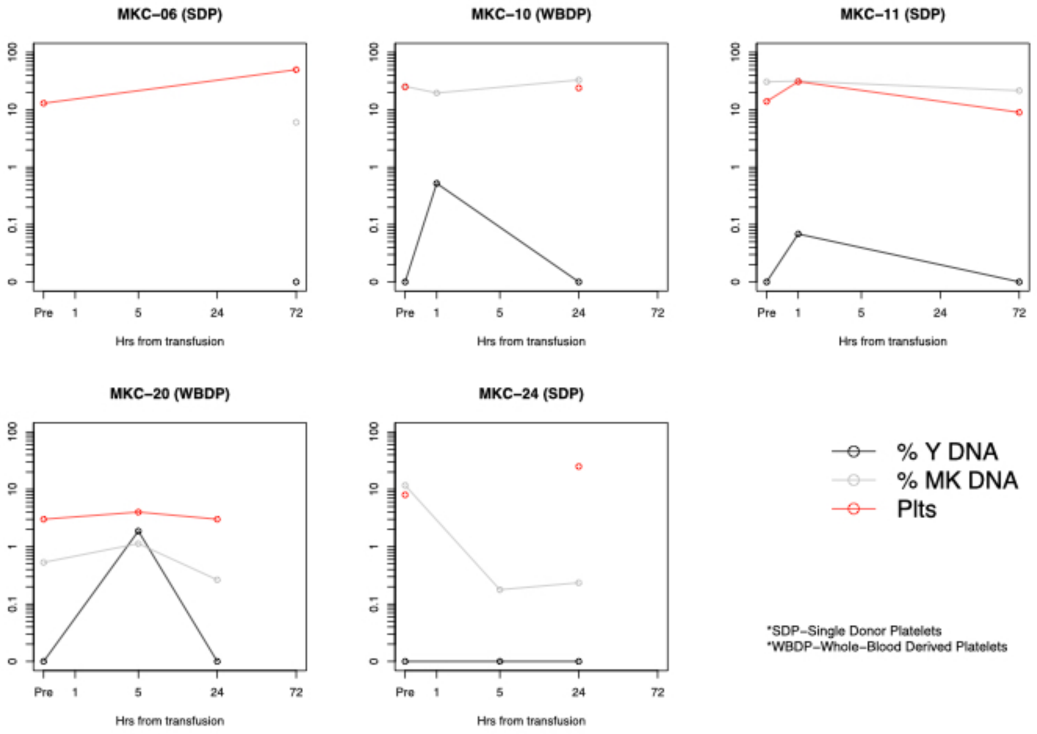

Figure S10. %Y chromosome DNA (SRY/b-Actin), platelet counts and MK DNA for females receiving male donor platelets. For 5 female individuals who received platelet transfusions from men, Y-chromosome DNA is eliminated from plasma by 24-72 hours, regardless of change in platelet levels.

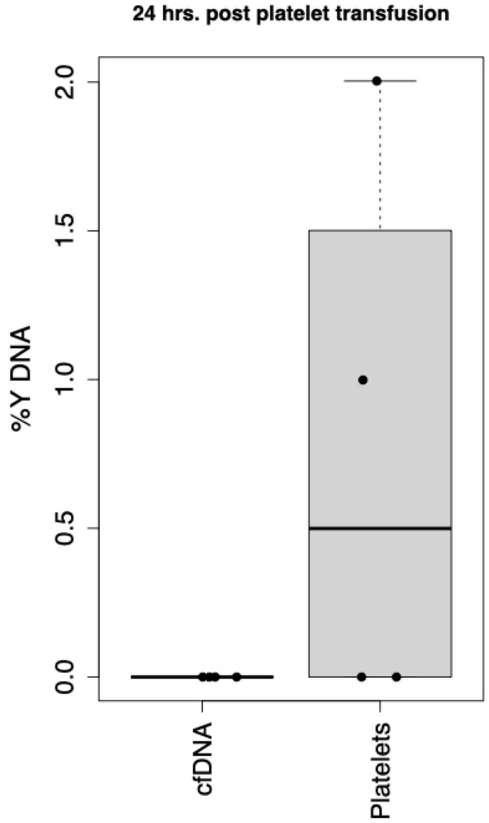

Figure S11. Y chromosome DNA in platelets. Percent Y chromosome DNA (SRY/b-Actin) was calculated in DNA of platelets and cfDNA of 4 women 24 hours after male platelet transfusion, demonstrating no Y chromosome DNA in cfDNA, despite presence in 2/4 cases of platelets.

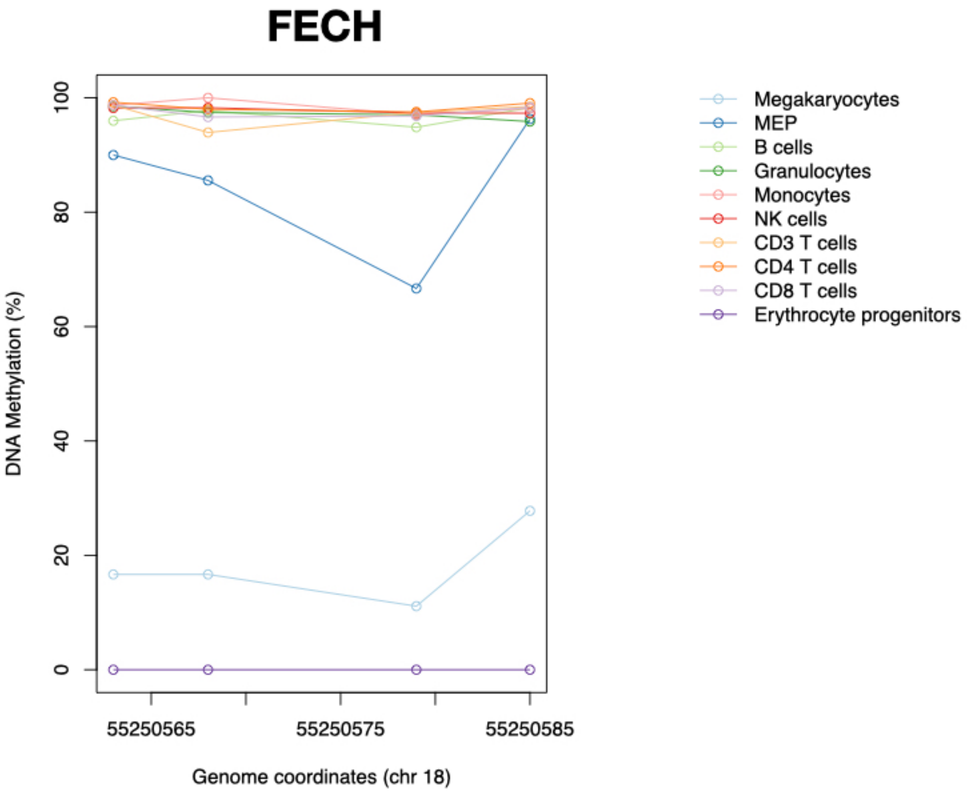

Figure S12. A previously reported erythroid-specific unmethylated marker is unmethylated in both erythroblasts and megakaryocytes. Percent DNA methylation at a previously published erythroblast-specific marker region[2] is analyzed using WGBS data at this region in multiple cell types, demonstrating that this marker is unmethylated both in erythroblasts and in megakaryocytes, suggesting that is not a specific marker to erythroblasts.

Supplementary References

1. Loyfer, N. *et al.* A DNA methylation atlas of normal human cell types. *Nat. 2023 6137943* **613**, 355–364 (2023).
2. Lam, W. K. J. *et al.* DNA of Erythroid Origin Is Present in Human Plasma and Informs the Types of Anemia. *Clin. Chem.* **63**, 1614–1623 (2017).
