# Supplementary material for: Megakaryocyte- and erythroblast-specific cell-free DNA patterns in plasma and platelets reflect thrombopoiesis and erythropoiesis levels
Source: Nat Commun. 2023 Nov 20;14:7542. doi: 10.1038/s41467-023-43310-2 (PMC10662131; doi:10.1038/s41467-023-43310-2)
Supplement: Supplementary file 3 — Reporting Summary [file 41467_2023_43310_MOESM3_ESM.pdf]

nature portfolio

Corresponding author(s): Prof. Yuval Dor

Last updated by author(s): Oct 25, 2023

Reporting Summary

Nature Portfolio wishes to improve the reproducibility of the work that we publish. This form provides structure for consistency and transparency in reporting. For further information on Nature Portfolio policies, see our Editorial Policies and the Editorial Policy Checklist.

Statistics

For all statistical analyses, confirm that the following items are present in the figure legend, table legend, main text, or Methods section.

n/a	Confirmed	
☐	☒	The exact sample size (*n*) for each experimental group/condition, given as a discrete number and unit of measurement
☐	☒	A statement on whether measurements were taken from distinct samples or whether the same sample was measured repeatedly
☐	☒	The statistical test(s) used AND whether they are one- or two-sided<br>*Only common tests should be described solely by name; describe more complex techniques in the Methods section.*
☐	☒	A description of all covariates tested
☐	☒	A description of any assumptions or corrections, such as tests of normality and adjustment for multiple comparisons
☐	☒	A full description of the statistical parameters including central tendency (e.g. means) or other basic estimates (e.g. regression coefficient) AND variation (e.g. standard deviation) or associated estimates of uncertainty (e.g. confidence intervals)
☐	☒	For null hypothesis testing, the test statistic (e.g. *F*, *t*, *r*) with confidence intervals, effect sizes, degrees of freedom and *P* value noted<br>*Give P values as exact values whenever suitable.*
☐	☒	For Bayesian analysis, information on the choice of priors and Markov chain Monte Carlo settings
☐	☒	For hierarchical and complex designs, identification of the appropriate level for tests and full reporting of outcomes
☐	☒	Estimates of effect sizes (e.g. Cohen's *d*, Pearson's *r*), indicating how they were calculated

Our web collection on statistics for biologists contains articles on many of the points above.

Software and code

Policy information about availability of computer code

Data collection	No software was used.
Data analysis	Whole genome bisulfite sequencing analysis was performed using wgbstools (V 0.1.0) (https://github.com/nloyfer/wgbs_tools). Targeted bisulifte sequencing analysis was performed using btseq (https://github.com/Joshmoss11/btseq).

For manuscripts utilizing custom algorithms or software that are central to the research but not yet described in published literature, software must be made available to editors and reviewers. We strongly encourage code deposition in a community repository (e.g. GitHub). See the Nature Portfolio guidelines for submitting code & software for further information.

Data

Policy information about availability of data

All manuscripts must include a data availability statement. This statement should provide the following information, where applicable:

- Accession codes, unique identifiers, or web links for publicly available datasets
- A description of any restrictions on data availability
- For clinical datasets or third party data, please ensure that the statement adheres to our policy

Source data are provided with this paper.
Results of targeted bisulfite sequencing for erythroblast and megakaryocyte methylation markers are included in Supplementary Table S3.
The whole-genome bisulfite sequencing (WGBS) data of platelet DNA generated in this study have been deposited in the GEO database under accession code

GSE206818. The raw sequencing data are protected and are not available due to data privacy laws. The targeted bisulfite sequencing data generated in this study are provided in the Supplementary Information/Source Data file. The bone-marrow residing progenitor cells WGBS data used in this study are available in the IHEC data portal. WGBS data for erythroblasts, leukocytes, hepatocytes, endothelial cells used in this study are available in the GEO database under accession code GSE186458.

Research involving human participants, their data, or biological material

Policy information about studies with human participants or human data. See also policy information about sex, gender (identity/presentation), and sexual orientation and race, ethnicity and racism.

Reporting on sex and gender	Self-reported sex was collected for all particpants constenting to share this information. In order to maintain relevant sample sizes for analyses, statistical analyses were performed on males/females together. However, all sex data is made available in the source data. For the experiment involving sex-mismatched platelet transfusions, all recipients were female, in order to allow y chromosome DNA detection as a surrogate for donor DNA.
Reporting on race, ethnicity, or other socially relevant groupings	Individuals were not grouped by race, ethnicity or other socially relevant grouping.
Population characteristics	The population was characterised based on current hematological diagnoses, with each individual being a member of one of the following groups: Healthy, bone marrow suppressed, ITP, ET, Thallasemia.
Recruitment	Healthy participants were voluntarily recruited at dedicated centres. Individuals with disease were recruited by treating physicians. There is unlikely to be a selection bias, as the likelihood to volunteer a blood draw is not likely to be related to the tested variables.
Ethics oversight	The study protocol was approved by the Committee on Research Involving Human Subjects of the Hebrew University-Hadassah Medical School, Jerusalem, Israel

Note that full information on the approval of the study protocol must also be provided in the manuscript.

Field-specific reporting

Please select the one below that is the best fit for your research. If you are not sure, read the appropriate sections before making your selection.

☒ Life sciences ☐ Behavioural & social sciences ☐ Ecological, evolutionary & environmental sciences

For a reference copy of the document with all sections, see nature.com/documents/nr-reporting-summary-flat.pdf

Life sciences study design

All studies must disclose on these points even when the disclosure is negative.

Sample size	A large amount of healthy individuals were recruited in order to establish normal cfDNA megakaryocyte/erythroblast levels (n=62 and n=72 respectively), and for further comparison to pathological cases. Estimated levels of megakaryocyte/erythroblast cfDNA by genome were assumed based on genome wide analyses. Subsequently, based on the estimation that a consistent effect of approximately >50% change would exist between cases with pathological clotting disorders and controls, it was estimated that 5 samples per pathology would suffice. For thalassemia, as it was estimated that erythroblast levels would be very low in healthy individuals based on genome wide analyses, and very high in thalassemia, we sufficed with 3 samples.
Data exclusions	Healthy controls were excluded if platelet count was less than 150000 platelets/ul or greater than 500000 platelets/ul.
Replication	Biologial replicates were performed in order to ensure robustness of the results. Furthermore, analyses were performed in multiple batches by different researchers, yet findings were not concluded based on a specific batch.
Randomization	Participants were not randomized. Covariates were collected and analyzed ad-hoc to ensure fair comparisons between groups.
Blinding	The investigators performing the experiments (DNA extraction, PCR, sequencing...) were blinded as to the group of the participants being analyzed.

Reporting for specific materials, systems and methods

We require information from authors about some types of materials, experimental systems and methods used in many studies. Here, indicate whether each material, system or method listed is relevant to your study. If you are not sure if a list item applies to your research, read the appropriate section before selecting a response.

Materials & experimental systems

n/a	Involved in the study
☒	☐ Antibodies
☒	☐ Eukaryotic cell lines
☒	☐ Palaeontology and archaeology
☒	☐ Animals and other organisms
☒	☐ Clinical data
☒	☐ Dual use research of concern
☒	☐ Plants

Methods

n/a	Involved in the study
☒	☐ ChIP-seq
☒	☐ Flow cytometry
☒	☐ MRI-based neuroimaging

Plants

Seed stocks

Report on the source of all seed stocks or other plant material used. If applicable, state the seed stock centre and catalogue number. If plant specimens were collected from the field, describe the collection location, date and sampling procedures.

Novel plant genotypes

Describe the methods by which all novel plant genotypes were produced. This includes those generated by transgenic approaches, gene editing, chemical/radiation-based mutagenesis and hybridization. For transgenic lines, describe the transformation method, the number of independent lines analyzed and the generation upon which experiments were performed. For gene-edited lines, describe the editor used, the endogenous sequence targeted for editing, the targeting guide RNA sequence (if applicable) and how the editor was applied.

Authentication

Describe any authentication procedures for each seed stock used or novel genotype generated. Describe any experiments used to assess the effect of a mutation and, where applicable, how potential secondary effects (e.g. second site T-DNA insertions, mosiacism, off-target gene editing) were examined.
